# Transcriptomes of newly-isolated *Trypanosoma brucei rhodesiense* reveal hundreds of mRNAs that are co-regulated with stumpy-form markers

**DOI:** 10.1186/s12864-015-2338-y

**Published:** 2015-12-29

**Authors:** Julius Mulindwa, Clémentine Mercé, Enock Matovu, John Enyaru, Christine Clayton

**Affiliations:** Department of Biochemistry and Sports Science, College of Natural Sciences, Makerere University, P.O.Box 7062, Kampala, Uganda; Zentrum für Molekulare Biologie der Universität Heidelberg, DKFZ-ZMBH Alliance, Im Neuenheimer Feld 282, D-69120 Heidelberg, Germany; Department of Biotechnical and Diagnostic Sciences, College of Veterinary Medicine, Animal Resources and Biosecurity, Makerere University, P.O.Box 7062, Kampala, Uganda

## Abstract

**Background:**

During natural *Trypanosoma brucei* infections, the parasites differentiate spontaneously into a non-dividing “stumpy” form when a certain level of parasitaemia is attained. This form is metabolically adapted for rapid further differentiation into procyclic forms upon uptake by Tsetse flies.

**Results:**

We describe here four central Ugandan isolates of *Trypanosoma brucei rhodesiense* that have undergone only three rodent passages since isolation from human patients. As expected, SNP analysis shows that these isolates are more closely related to each other than to the commonly used strains Lister 427, Antat1.1, and TREU927. TREU927 generally has smaller copy numbers of repeated genes than the other strains, while Lister 427 trypanosomes with a 30-year history of in vitro culture and cloning have more histone genes than the other isolates. The recently isolated trypanosomes were grown in rats, and their transcriptomes characterised. In comparison with cultured procyclic and bloodstream forms, there were increases in mRNAs encoding the stumpy-form markers ESAG9 and PIP39, with coordinated alterations in the levels of over 600 additional mRNAs. Numerous mRNAs encoding proteins of no known function were either increased or decreased. The products of the mRNAs that were increased in parallel with *PIP39* included not only enzymes of procyclic-form metabolism, but also components of the translational and RNA control machineries. Many of the mRNAs that were decreased in cells with elevated *PIP39* reflected reduced cell division.

**Conclusions:**

These transcriptomes suggest new avenues for research into the regulation of trypanosome differentiation.

**Electronic supplementary material:**

The online version of this article (doi:10.1186/s12864-015-2338-y) contains supplementary material, which is available to authorized users.

## Background

Salivarian trypanosomes are protist parasites which infect mammals and Tsetse flies and are extracellular throughout the life cycle. *Trypanosoma brucei* subspecies multiply in the blood and tissue fluids of mammals. *Trypanosoma brucei brucei* infects cattle and other mammals, but is killed by trypanolytic factors found in the serum of human and some old world apes [1]. Disease in humans is caused by *Trypanosoma brucei rhodesiense* and *Trypanosoma brucei gambiense*, both of which are resistant to the trypanolytic factors. *T. b. rhodesiense* differs from *T. b. brucei* only in that the former has an additional gene, called SRA, which mediates serum resistance [2].

*T. brucei* multiplies in mammals as long slender trypomastigotes. The long slender form has a very reduced mitochondrion, relying on glucose and substrate-level phosphorylation for ATP generation and variant surface glycoprotein for defence against humoral immunity. In contrast, the procyclic form, which is found in the Tsetse midgut, has a more elaborate mitochondrion and, on its surface, two repetitive proteins called GPEET and EP procyclin. Both bloodstream and procyclic forms are readily cultured in vitro. Another form is, however, present in the blood - the “stumpy” form. Stumpy forms are - as the name implies - relatively shorter and fatter than long slender forms, and have a slightly more elaborate mitochondrion with some cytochrome development [3]. Stumpy forms are “pre-adapted” for procyclic differentiation [4]. They are arrested at the G_0_ stage of the cell cycle and can only resume dividing if placed into conditions conducive to development of procyclic forms.

When mice are infected with trypanosomes that can undergo stumpy differentiation (“pleomorphic” lines), there is an initial rise in parasites, which flattens out as stumpy differentiation occurs; trypanosomes then disappear from the blood as an immune response develops to the variant surface glycoprotein (VSG). After a few days, new parasites with a different VSG appear in the blood. The result is a fluctuating parasitaemia, which can last for several weeks before the immune response of the animals is exhausted. To obtain pure stumpy-form populations, pleomorphic *T. b. brucei* are grown in immunosuppressed mice [5] to densities of about 2–5 × 10^8^/ml [6, 7], but even without immunosuppression, most cells will be stumpy at peak parasitaemia [8]. One unexplained dichotomy is that in vitro, the same cell lines undergo growth arrest at the much lower density of 10^6^ cells/ml [9].

Continuous passage of bloodstream-form *T. brucei* in either mice or culture strongly selects against the ability to complete stumpy differentiation. Many experiments are done using the resulting “monomorphic” lines, which have the advantage of growing fast and to densities up to 7 ×10^6^/ml; this includes most cultures of the Lister 427 line. In general, mice infected with parasites that cannot make stumpy forms develop fulminating, non-resolving parasitaemias, unless the inoculum is very low.

Stumpy formation is triggered by a quorum-sensing mechanism: pleomorphic trypanosomes secrete, and also respond to, an as-yet-unidentified soluble factor [9]. The stumpy differentiation process can also be induced by addition of cell-permeable molecules which cause an increase in internal AMP [10]. An RNA interference screen identified about 30 genes whose expression is required for the response to increased AMP; apart from genes involved in AMP metabolism, they include a variety of potential regulators [10]. A protein tyrosine phosphatase called PTP1 inhibits differentiation of stumpy forms to procyclics [11] by inactivating a second phosphatase called PIP39 [12].

Two groups have investigated gene expression changes during differentiation of long slender trypomastigotes to stumpy forms, then procyclic forms using microarrays [6, 7]; Capewell et al. [13] extended the analysis of stumpy differentiation to polysomal RNA. All studies identified PIP39 among the mRNAs that had increased abundance in stumpy forms. Other stumpy markers are the surface receptor PAD1 [14] and the secreted protein ESAG9 [15]. Use of ESAG9 as a stumpy marker is, however, complicated by the existence of numerous paralogues, which vary between trypanosome isolates. Using PAD1 as a marker, MacGregor et al. [8] showed that in chronic mouse infections with pleomorphic trypanosomes, stumpy forms initially appear in the blood when parasitaemias exceed 10^8^/ml. After careful measurements through infection, and modeling, the authors concluded that the tendency of a parasite strain to make stumpy forms is a very important factor in determining the level of parasitaemia [8].

All transcriptome analyses published so far have been done using trypanosomes that have been in the laboratory for over 30 years. We recently isolated *T. b. rhodesiense* from Ugandan sleeping sickness patients. We compared the transcriptomes of these parasites, grown in intact rats with only a single intermediate passage, with that of cultured monomorphic trypanosomes grown in culture. RNA preparations from two of the recent isolates had high levels of mRNAs encoding stumpy markers, and significant differences in expression of over 600 other genes, compared with the cultured cells.

## Methods

### Trypanosome samples and infections

Patient recruitment was carried out at Lwala hospital in Kaberamaido district, which is situated in the relatively new *T. b. rhodesiense* human African trypanosomiasis focus in North-Eastern Uganda. Diagnosis was by microscopic detection of motile trypanosomes on a wet blood smear of finger prick blood.

Ethical approval of protocols was obtained from the Ministry of Health and Uganda National Council of Science and Technology (UNCST), Uganda, and the ethics committee of University of Heidelberg, Germany. All patients recruited into this study received written and verbal information explaining the purpose of the study and they gave informed consent. The ethical consent forms were written in English and translated into the local languages. For the children and adolescent participants (below 18 years), parents or guardians gave informed consent on their behalf. Peripheral blood (3–4 ml) from confirmed Human African Trypanosomiasis patients was collected into an EDTA tube (BD Vacutainer) upon phlebotomy. An aliquot of the whole blood (600 μl) was cryopreserved, a drop (10 μl) was spotted on Whatman paper for PCR diagnosis. In order to confirm that all the cases were *T. b. rhodesiense* infections, PCR was carried out on the SRA gene as described in [16].

To follow mouse infections, a stabilate was thawed and injected into a mouse. Once these parasitaemias had attained about 5 × 10^7^ trypanosomes/ml, 2-week-old inbred Swiss white mice were infected with 500 parasites each. All infections were done at the same time. Parasites were counted by diluting 10 μL of tail blood into 1 mL of phosphate-saline-glucose, then counting in a haemocytometer. Mice were killed if obvious terminal symptoms were observed.

For RNA preparation, rats were infected with 5000 parasites each. Parasites were followed using wet blood films, and parasitaemias were estimated by the matching method [17]. After 7 days, blood (3–4 mL) was collected by cardiac puncture into an EDTA tube. After centrifugation at 3000 × g for 10 min, the plasma was aspirated off and the buffy coat layer (approx. 500 μl) carefully resuspended without disturbing the underlying more compact layer of leucocytes. The buffy coat was transferred to a 1.5 ml microfuge tube, centrifuged at 6000 rpm for 2 min at room temperature in the microfuge and the pellet fraction resuspended in 1 ml of Trifast reagent (Peqlab, GmbH) for RNA preparation.

### Sequence analysis

Trypanosomes were cultured exactly as described in [18]. Sequencing of genomic DNA and poly(A)+ RNA was done using standard Illumina kits. The sequences were aligned using Bowtie or BWA and sorted and indexed using SAMtools [19, 20]. Reads aligning to open reading frames of the TREU927 genome were counted using a custom python script. The transcriptome data were analysed in R, using (in part) the DESeq package [21]. Pattern analysis was done using a custom R script and category enrichment calculated using the Fisher exact test.

To obtain copy numbers, we calculated reads per million per kilobase for all open reading frames. The result was a continuous distribution, with a strong peak and a tail towards higher values (Additional file 1: Figure S1). For each dataset we chose the modal RPKM value to represent a single-copy gene, and divided the other values by this to attain gene copy numbers, as described [18]. For SNP and indel analysis, duplicated reads were removed using picard and then realignment around indels was carried out using GATK [22]. Variant calling was performed using Samtools mpileup with the read depth cutoff of 100. SNP and INDEL tagging was then done using snpEff into a vcf file.

## Results

### The four new isolates cause chronic infections in mice

**Fig. 1:**
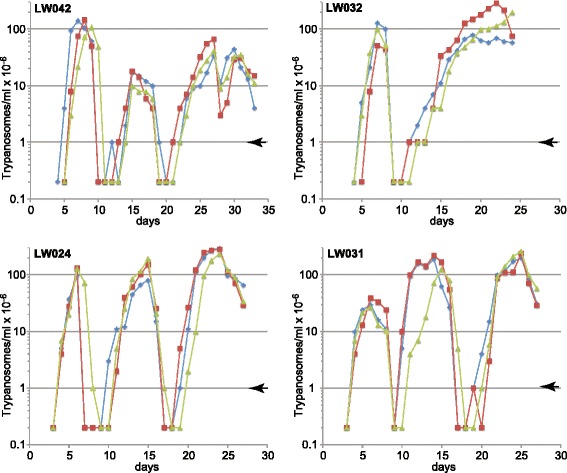
Parasitaemias from the four new isolates in mice. For each isolate, three mice (male for LW042 and LW032, female for LW024 and LW031) were injected with parasites from fresh mouse blood, and parasites were counted. The parasitaemias of the individual mice are shown. The arrows on the right indicate the threshold of detection

New *T. rhodesiense* isolates from human patients were tested for infectiveness for mice. Four isolates that readily infected mice on first passage were used to re-infect mice and the parasitaemias were followed. As expected, the new isolates caused fluctuating parasitaemias, which were fatal after 3–5 weeks. All four lines showed similar maximal parasitaemias of 2–3 × 10^8^/ml, suggesting that they have similar quorum sensing thresholds (Fig. 1). Although some differences between the parasitaemias were observed, we do not know whether these are intrinsic to the different isolates: for example, it is also possible that there were differing numbers of infectious parasites (not committed to stumpy formation) in the inoculates.

### The genomes of four new isolates: copy number variations and sequence variations

**Fig. 2:**
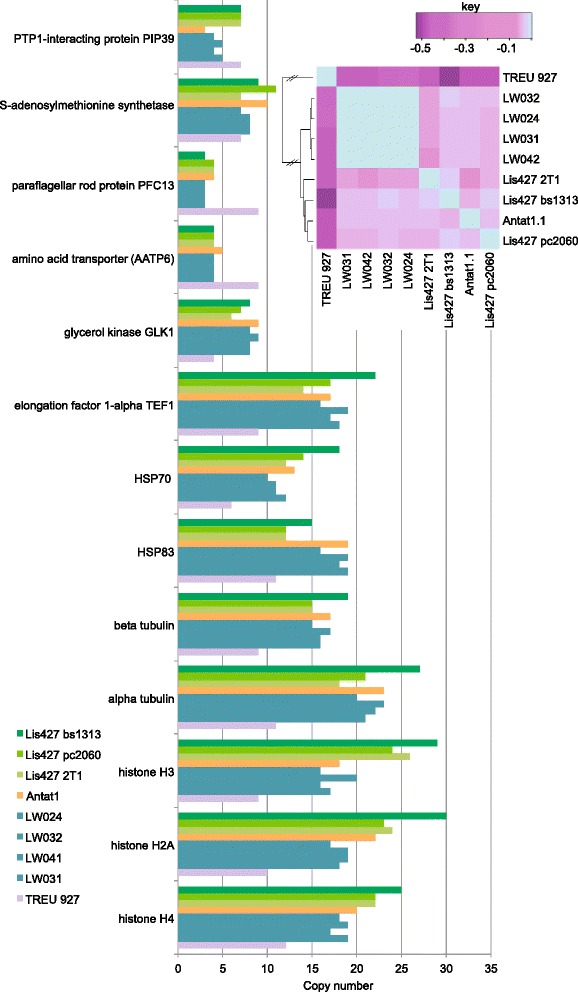
Copy numbers of selected genes, deduced from shotgun sequencing

The genomes of all four isolates, our normal lab Lister 427 trypanosomes, and cultured Antat1.1 bloodstreams forms were shotgun sequenced, and analysed for copy number variations and single nucleotide polymorphisms. Coverage was approximately 30-fold. The original reads for TREU927 were used for comparison. Copy number analysis for all genes is in Additional file 2: Table S1. The TREU927 strain was initially chosen for sequencing because of its small genome, and low numbers of repeats were evident for several multi-copy genes (Fig. 2). Three different clones of Lister 427 expressing the *tet* repressor were compared; these had a slight excess of histone genes, perhaps reflecting long years of selection for fast growth in culture (Fig. 2). It would be interesting to investigate the copy-numbers in earlier, less-passaged stocks of Lister 427. A differentiation-competent AnTat1.1 isolate was intermediate in copy numbers for these particular genes. Including all genes, comparison of the new isolate data with the reference genome revealed about 24,000 indels and 150,000–200,000 SNPs, slightly over 50 % of which were homozygous (Additional file 3: Table S2). However, when large gene families such as those encoding Variant Surface Glycoproteins and Expression Site Associated Genes were removed, and the analysis was restricted to a single-copy open reading frames in the reference, the number of SNPs was reduced to 50,000. SNP analysis for the Lister427 and AnTat1 genomes revealed similar levels of deviation from the reference. We used the copy numbers to construct an evolutionary tree. Both this (Fig. 2, inset) and phylogenetic analysis of the SNPs (not shown) showed, as expected, that the four Ugandan strains were more closely related to each other than to the other strains. The copy numbers of our repressor-expressing cultured bloodstream-form and procylic-form Lister 427 differed somewhat; this is not really surprising since these lines had been cultured separately for at least 25 years, and had also been transfected with plasmids and cloned.

### Some of the recently-isolated trypanosome transcriptomes show high levels of mRNAs encoding stumpy-form markers

**Fig. 3:**
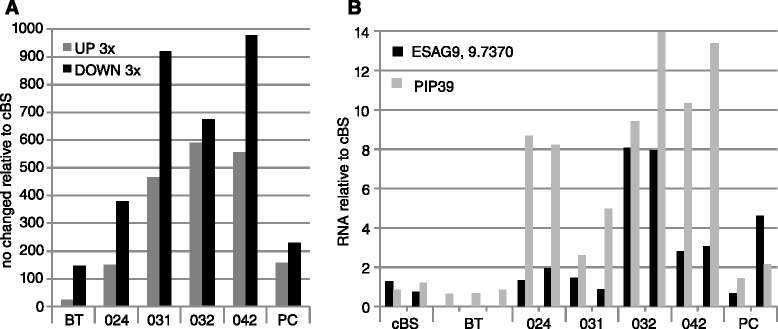
Comparison of transcriptomes of pleomorphic bloodstream forms with laboratory-adapted trypanosomes. **a** Number of genes showing differences in expression, relative to cultured bloodstream-form Lister 427 parasites at 1 × 10^6^ /mL. BT: data from three rat infections as described in [23]. PC: Procyclic-form Lister 427 parasites at no more than 2.5 × 10^6^ /mL. All results are for poly(A)+ mRNA, and only one representative of each gene family is included. We selected genes with at least 3-fold regulation, and an adjusted p-value threshold of 0.001. **b** Relative levels of mRNA (reads per million reads) from *PIP39* and the *ESAG9* locus Tb 927.9.7370 in the different samples

We next obtained transcriptomes from the four isolates. Each was used to infect two rats, and the parasites were allowed to grow for 7 days, by which time the parasitaemias were estimated at between 2 × 10^8^/ml and 6 × 10^8^/ml [17]. We prepared RNA from buffy-coat parasites with minimal handling, since purification changes trypanosome transcriptomes [23]. Replicate RNA samples were analysed by high-throughput cDNA sequencing (RNASeq). Approximately 70 % of the reads aligned to the trypanosome genome (12–30 million reads). Initial analysis showed that the transcriptomes of replicates correlated best with each other (Additional file 4: Table S3, sheet 1). We compared them with previously published results for cultured *T. b. brucei* Lister 427 bloodstream forms and procyclic forms [24–27], and for lab-adapted *T. b. rhodesiense* (strain Tbr729) grown in rats [23]. Even when strict criteria were applied (Padj <0.001, >3-fold difference in expression) (Additional file 5: Table S4), the numbers of “significantly regulated” genes in the new isolate transcriptomes were striking (Fig. 3a). Notably, there was clear up-regulation of the *PIP39* mRNA, especially in the LW032 and LW042 samples, but also to some extent in the others (Fig. 3b, Additional file 4: Table S3, sheet 5). In addition, there were increases in reads that mapped to *ESAG9* genes, although the pattern was more heterogeneous (Fig. 3b), presumably due to inter-strain differences in *ESAG9* gene content, as well as use of different VSG expression sites (which contain *ESAG9* genes) (Additional file 4: Table S3, sheet 5). PAD1 is a member of a multi-gene family. Read counts for the PAD family as a whole were increased in LW032 and LW042 transcriptomes (Additional file 4: Table S3, sheet 5) but we cannot tell which paralogue they came from. These expression profiles suggested that the trypanosome samples from blood contained mixtures of trypanosomes at various stages of stumpy-form differentiation, with least stumpy-form expression in the Tbr729 sample, more in LW031 and LW024, and most in LW032 and LW042. The mRNAs that were most strongly regulated in LW032 and LW042 may therefore be stumpy-specific. This designation is however very tentative: although the parasitaemias in all rats were comparable with those seen at the first peak in mice, they were not counted accurately and we did not assess trypanosome morphology before RNA preparation.

**Fig. 4:**
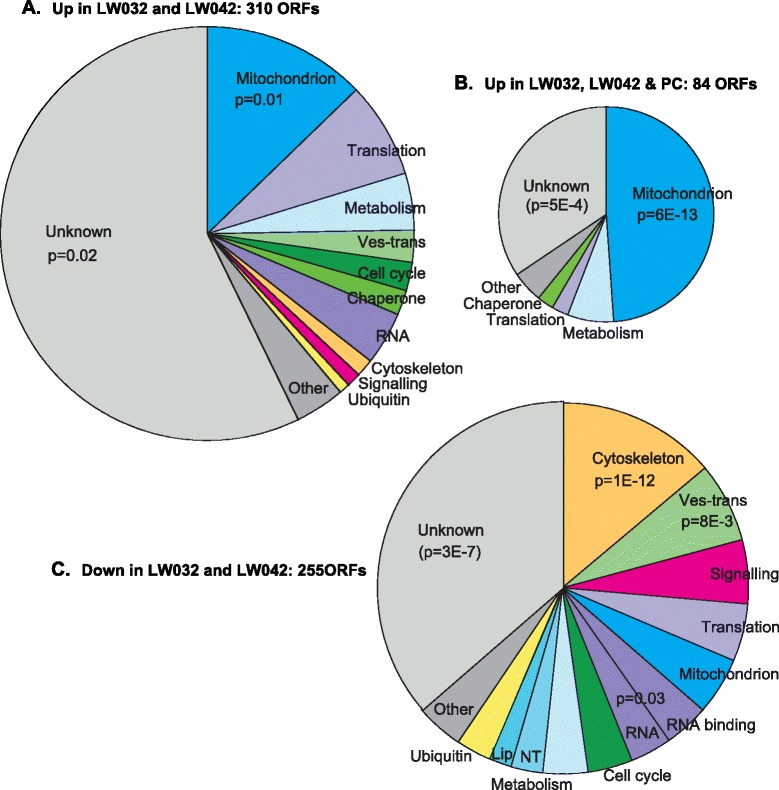
Functional annotation of genes showing differential expression in the LW042 and LW032 transcriptomes. The datasets are from Additional file 5: Table S4. The numbers of genes are indicated and the circle areas are proportional to those gene numbers. Probability values are results from the Fischer exact test; categories that are under-represented are shown in parentheses and italics. **a** Increased relative to cultured bloodstream and procyclic forms, and Tbr729 bloodstream forms. Ves-trans: vesicular transport and lysosome; Ubiquitin: ubiquitination and proteasome; RNA: RNA-binding proteins and other RNA processes. **b** Increased relative to cultured Lister 427 bloodstream forms, and Tbr729 bloodstream forms; also increased in procyclics. **c** Decreased relative to cultured bloodstream and procyclic forms, and Tbr729 bloodstream forms. *Ves-trans* vesicular transport and lysosome, *Ubiquitin* ubiquitination and proteasome, *RNA* RNA processes apart from binding proteins, *Lip* enzymes of lipid and fatty acid metabolism, *NT* nucleotide metabolism

Because LW032 and LW042 had the highest *PIP39* expression, we searched for mRNAs that showed significantly higher expression in those two lines compared with Tbr729 bloodstream forms, cultured Lister 427 bloodstream forms, and procyclic forms. This yielded a list of 310 genes whose mRNAs were significantly increased (Additional file 5: Table S4, sheet 2). Most of these mRNAs showed somewhat higher expression in LW031, a little more in LW024, and a peak in either LW042 or LW032 (Additional file 6: Figure S2). Comparison with previous transcriptome results for stumpy forms [6, 7, 13] showed that 135 of these mRNAs had been identified as being increased at least 1.5-fold in stumpy forms, relative to cultured bloodstream forms, in at least one previous study. Over the whole dataset, however, there was no correlation - perhaps because the previous experiments had used less sensitive methods. Intriguingly, genes encoding proteins of unknown function dominated the list (Fig. 4a). In a published RNAi screen [28], 16 of these were identified as being required for procyclic differentiation (Additional file 5: Table S4, sheet 2). There were also many up-regulated genes encoding mitochondrial proteins, including ribosomal proteins and cytochromes.

We also looked at mRNAs that were increased in LW032 and LW042 transcriptomes, and stayed high in procyclic forms (Fig. 4b, Additional file 5: Table S4, sheet 3). These were enriched in mitochondrial proteins, and also included other enzymes implicated in procyclic metabolism. LW032 and LW042 showed increases in mRNAs encoding two uncharacterised protein phosphatases (Tb927.9.3470 and Tb92710.4930) and the RNA-binding protein RBP34. *ZC3H11* mRNA was also elevated. ZC3H11 is involved in the heat shock response [26], so its mRNA could have been increased either as part of differentiation, but it is also possible that the rats had fever (this was not measured). Some changes might have been correlated with cell cycle arrest: we saw up-regulation of the mRNAs encoding subunit 10 of the anaphase promoting complex, cyclin 7, and kinetochore protein 16.

Rather surprisingly, 20 cytosolic ribosomal protein mRNAs also specifically increased in the LW042 and LW032 transcriptomes. Stumpy forms have very little translation compared with long slender forms [29] so should actually have lower ribosomal protein requirements. Perhaps these mRNAs, too, are not being well translated. Indeed, a previous analysis of stumpy-form polysomes, done using a sequence-tagging approach [13], indicated that ribosomal protein mRNAs are mostly rather poorly represented in the polysomal fraction.

Salivary gland trypanosomes contain a high proportion of metacyclics, which, like stumpy forms, are non-dividing. A comparison of their transcriptomes [30] with those of bloodstream and procyclic forms revealed 126 increased mRNAs that were also elevated in the LW032 and LW042 transcriptomes (Additional file 5: Table S4, sheet 6). These included the two cell-cycle RNAs mentioned above, and mRNAs encoding the RNA-binding protein RBP5, a protein kinase and two chaperones.

### High stumpy-form marker expression is accompanied by reduced expression of genes required for cell proliferation

Stumpy forms are expected to show decreases in mRNAs required for cell proliferation. Of the 253 mRNAs that were significantly decreased in the LW032 and LW042 transcriptomes (Additional file 5: Table S4, sheet 4), 41 (16 %) showed cell-cycle variation with an amplitude of at least 1 [31]. An amplitude of 1 means that highest expression measured was at least twice the lowest value. The 16 % proportion is a significant enrichment (*p* = 3E-15) over the ~4 % of genes that are similarly regulated in the whole gene set. There was significant enrichment for all cell-cycle stages, but particularly for mRNAs that are normally increased in late G1 (*p* = 1E-7) and S-phase (*p* = 5E-8).

Not unexpectedly, the reduced gene products were enriched in cytoskeletal proteins, required for formation of new flagellae (Fig. 4c, Additional file 5: Table S4, sheet 4). There were also several protein kinases (including TOR1 and TOR3), and protein phosphatases in the list, four kinetochore proteins, two nuclear DNA repair enzymes, 8 proteins involved in kDNA replication, the histone variant H2AZ, a few ubiquitination pathway proteins, various components of the vesicular transport machinery, and some factors required for RNA processing. The mRNAs for translation elongation factor EF2 and the translation initiation helicase eIF4A1, and some tRNA synthetases, also had reduced abundance relative to cultured bloodstream and procyclic forms. Rather intriguingly, two homologues of the cap-binding initiation complex, eIF4E2 and eIF4G2, also showed decreases: there is no evidence that these are involved in translation and their function is unknown. There was slight enrichment for proteins involved in RNA metabolism; mRNAs encoding proteins with RNA-binding domains included the cell cycle binding sequence protein CSBP2, DRBD14, DRBD17, DRBD18, RBP10, RBP26, ZC3H12, ZC3H41 and ZC3H46 and Tb927.11.3500. For all of these genes, there was a gradation in expression that was the inverse of that seen in the up-regulated set (Additional file 7: Figure S3). DRBD18 - which regulates many mRNAs [32] - and RBP26 mRNAs are also decreased in salivary gland trypanosomes [30]. 47 mRNAs were at least 2.5-fold down-regulated in both LW032 and LW042 and metacyclics (Additional file 5: Table S4, sheet 7), with reductions of no fewer than 8 mRNAs encoding RNA-binding proteins.

The set of genes in Additional file 5: Table S4, sheet 4 was selected to have decreased expression relative to the *T. rhodesiense* strain Tbr729 bloodstream-forms as well as the cultured bloodstream forms, but these two datasets were not identical. This is not really surprising: the chemical environments are different, and the cultured bloodstream forms were carefully harvested during exponential growth (density less than 1.5 × 10^6^/ml), whereas the *T. rhodesiense* strain Tbr729 bloodstream-forms were harvested from blood at much higher densities. Twenty-three mRNAs were over-represented in cultured bloodstream forms only (Additional file 5: Table S4, sheet 5). These included mRNAs encoding 5 RNA-binding proteins: for example HNRNPH, which has roles in both mRNA stability and splicing [33], and RBP10, whose expression correlates with that of mRNAs encoding enzymes of bloodstream-form energy metabolism [34].

Nearly 100 genes showed at least 3-fold less mRNA in all of the blood trypanosomes relative to cultured bloodstream- or procyclic-form Lister 427 (Additional file 5: Table S4, sheet 6). They included *PUF9*, the product of which stabilises S-phase mRNAs, and one of its target mRNAs, *PNT1* [35]. The mRNA for the mitotic cyclin CYC6 was reduced in the trypanosomes from blood, in salivary gland trypanosomes and also, interestingly, in metacyclic *L major*, which are non-dividing [36]. Many of the RNAs that were less abundant in blood trypanosomes than in cultured ones were most decreased in the LW032 and LW042 cell populations, which suggests that these may be mRNAs that show very early reductions upon density sensing. Twenty-two of them were also under-represented in salivary gland trypanosome RNA; these included *PUF2*, *PUF4*, *ZC3H8*, *PIE8* and several encoding proteins implicated in signal transduction.

## Discussion

The transcriptomes described here reveal hundreds of mRNAs whose expression levels were significantly decreased or increased in cells that had an elevated amount of *PIP39* mRNA. The transcriptome profiles are consistent with the biology of stumpy forms, with decreases in mRNAs required for cell proliferation, and increases in mRNAs encoding proteins of procyclic-form metabolism. It is well known that trypanosomes that have recently been transmitted naturally undergo stumpy differentiation at parasitaemias above 10^8^/ml, and our parasites were all harvested at parasitaemias above that threshold. These datasets may therefore illustrate the progressive changes that are seen during the development of stumpy forms. It will be interesting to compare these results with those from laboratory strains that have been characterised in detail with respect to cell shape, cell cycle stage, and expression of suitable markers. If it turns out that the transcriptomes of the recent isolates show more regulation than those of lab strains, it would make sense to investigate the recent isolates in more detail.

An increase in mRNA does not necessarily mean that the protein is being made. Results of ribosome profiling [37, 38] and polysome analyses [13] demonstrate extensive regulation of translation. Since stumpy forms have low overall translation activity [29], it is possible that many of the mRNAs that had accumulated in the LW042 and LW032 cell populations were being stored in a translationally inactive form, ready for rapid activation upon uptake by a Tsetse fly. To confirm that regulation of any specific gene correlates with stumpy development, the ideal procedure will be to analyse expression of the encoded protein at the single-cell level, with counter-staining against a recognised marker such as PAD1.

Probably the most interesting differences that we saw were in potential regulators. The transcriptomes of the recently isolated trypanosomes showed changes in mRNAs encoding numerous RNA-binding proteins and in signal transduction proteins that have not hitherto been recognised as being involved in differentiation. The most interesting, however, may be the proteins whose functions have not yet been characterised at all.

## Conclusion

Comparison of our new transcriptomes with previous results, especially from functional screens, may suggest novel candidate regulators of trypanosome differentiation.

## Availability of supporting data

All results are available at Array Express. Raw reads for all *T. rhodesiense* transcriptomes, and two of the *T. brucei* transcriptomes, have accession number E-MTAB-3793. The raw data for the other two *T. brucei* transcriptomes have been lost but read counts are in the supplement. The bam files for all genomes mentioned are available from Array Express with accession number E-MTAB-3800.

## Ethics, consent and permissions

Work with human subjects was approved by the medical ethics committees of Heidelberg University and Makerere University.

Work with animals was approved by the Makerere University Animal use committee.

## Additional files

Additional file 1: Figure S1. Reads per Kilobase per million reads (RPKMs) for the set of unique open reading frames: examples from the genomic sequence data. Open reading frames were separated into “bins” according to the measured RPKM. The number of open reading frames in each bin was then calculated. The plots show the results for three of the genome datasets. The modal values were assumed to represent the average RPKM for a single-copy gene. (PDF 414 kb) Additional file 2: Table S1. Calculated gene copy numbers. Sheet 1: RPKM counts for all genes, modal values and calculated gene copy numbers. Sheet 2 Calculated gene copy numbers (RPKM divided by modal RPKM) for the unique gene list [39]. (XLS 3352 kb) Additional file 3: Table S2. SNPs and indels for the new isolates. The first sheet is a summary, the others are as labelled on the tabs. (XLSX 12654 kb) Additional file 4: Table S3. Raw poly(A)+ mRNA transcriptome data and calculations based on reads per million (RPM). Sheet 1: Alignment statistics and correlations between the different datasets, calculated from RPM using the unique gene set only. cBS: cultured Lister 427 bloodstream forms expressing the *tet* repressor from pHD1313. BT1-3: Tbr729; LW - new isolates; PC - cultured Lister 427 procyclic forms. Sheet 2: Read counts for all genes. Sheet 3: Data for unique gene list [39] with reads per million (RPM). Column R shows the regulation pattern according to a custom script. Samples giving significantly peak RPMs (relative to other samples) are indicated by “max” and troughs are indicated by “min”. The threshold was set at 2-fold. Sheet 4: Sheet 3 results, filtered to remove genes for which the maximum RPM value was less than 10. Column T shows correlation coefficients of the RPM results with *PIP39* (Tb927.9.6090). Colour code for ratios: Blue is less than 0.25×, green is less than 0.5×, orange is greater than 2× and red is greater than 4×. Comparisons with other datasets are indicated as follows: ST/SL Kabani; stumpy/slender ratio from [6]; ST/SL BS Jensen - stumpy/slender ratio from [7]; SLvST mRNA Capewell and ST polysomes/mRNA Capewell - data from [13]; SG/cBS: salivary gland trypanosomes relative to cultured bloodstream forms from [30]; SG/PC: salivary gland trypanosomes [30] relative to our results for cultured Lister 427 procyclic forms. Sheet 5: calculated RPMs for selected recognised stumpy-form markers. Reads for the *ESAG9*s and the *PAD* genes are not unique and precise allocation to a particular variant was not attempted. Sheet 6: Genes showing increased RPM values in both LW032 and LW042 and metacyclic (salivary gland, SG) forms. Sheet 7: Genes showing reduced RPM values in both LW032 and LW042 and metacyclic (salivary gland, SG) forms. (XLSX 3928 kb) Additional file 5: Table S4. Transcriptome analysis using DESeq. Sheet 1: Using only the unique gene set, transcriptomes from different isolates were compared pair-wise using DESeq. We show only the fold change and the adjusted P-values. Background colours are used only to make the pairs clear. Sheet 2: mRNAs that are most specifically increased in the LW032 and LW042 trasncriptomes relative to cBS, BT, and PC. Only genes with with adjusted P-values of less than 0.01 were considered. Of these, at least one isolate (either 42 or 32) had to show a 3-fold increase relative to cultured bloodstream forms, and the remainder of the ratios had to be at least 2× cBS or BT. B42 or 32 also had to be at least 2-fold procyclics. Comparisons with other datasets in this and subsequent sheets are indicated as follows: ST/SL Kabani; stumpy/slender ratio from [6]; ST/SL BS Jensen -stumpy/slender ratio from [7]; SLvST mRNA Capewell and ST polysomes/mRNA Capewell - data from [13]; SG/cBS: salivary gland trypanosomes relative to cultured bloodstream forms from [30]; SG/PC: salivary gland trypanosomes [30] relative to our results for cultured Lister 427 procyclic forms: cell cycle peak and amplitude: data from [31]. RITSeq: data from [28]. Sheet 3: mRNAs increased in procyclics as well as in LW032 and LW042. As for sheet 2 except that results for B42 and B32 had to be more than 0.5× the procyclic value. Sheet 4: mRNAs decreased at least two-fold in LW032 and LW042 relative to procyclics and bloodstream forms. Calculations similar to sheet 2. Sheet 5: mRNAs that were at least 3-fold higher in cBS relative to the other conditions. Sheet 6: mRNAs that were low in all parasites isolated from blood (down in BT as well as the recent isolates). (XLSX 4058 kb) Additional file 6: Figure S2. Heat map of the regulation factors (log2) for mRNAs that are increased in the LW032 and LW042 transcriptomes. Red is least and white is most. (PDF 617 kb) Additional file 7: Figure S3. Heat map of the regulation factors (log2) for mRNAs that are decreased in the LW032 and LW042 transcriptomes. Red is least and white is most. (PDF 570 kb)

## Abbreviations

VSG: variant surface glycoprotein
RNASeq: high-throughput cDNA sequencing
