# Supplementary figures and images for: Transcriptomes of newly-isolated Trypanosoma brucei rhodesiense reveal hundreds of mRNAs that are co-regulated with stumpy-form markers
Source: BMC Genomics. 2015 Dec 29;16:1118. doi: 10.1186/s12864-015-2338-y (PMC4696300; doi:10.1186/s12864-015-2338-y)

Antat 1.1

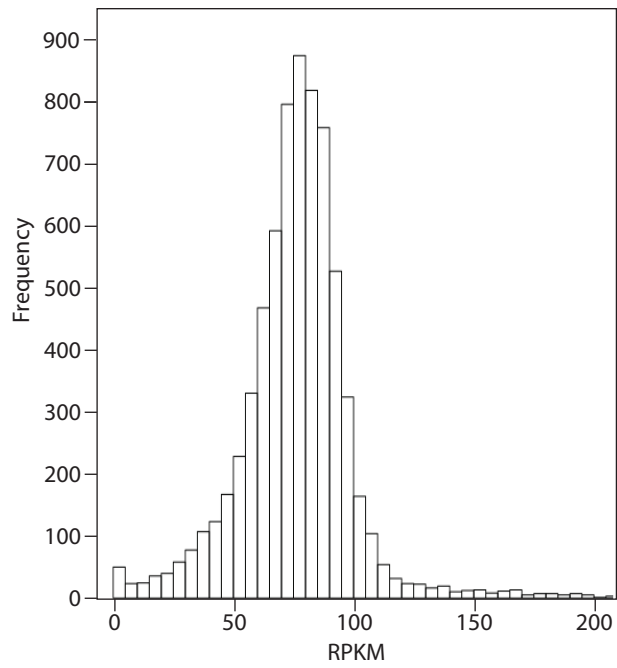

Lister 427 - 1313

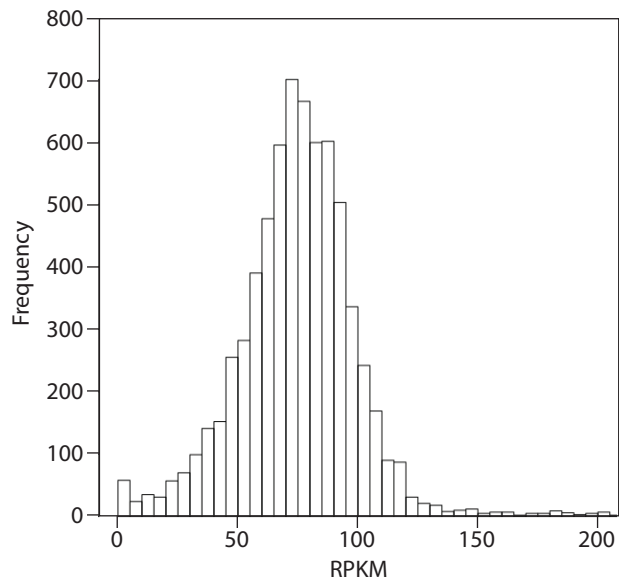

LW024

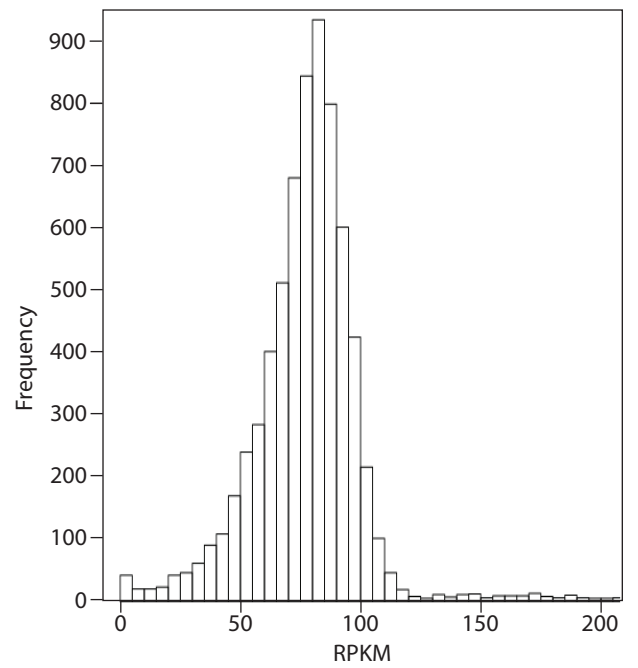

Supplement: Additional file 1: Figure S1. — Reads per Kilobase per million reads (RPKMs) for the set of unique open reading frames: examples from the genomic sequence data. Open reading frames were separated into “bins” according to the measured RPKM. The number of open reading frames in each bin was then calculated. The plots show the results for three of the genome datasets. The modal values were assumed to represent the average RPKM for a single-copy gene. (PDF 414 kb) [file 12864_2015_2338_MOESM1_ESM.pdf]

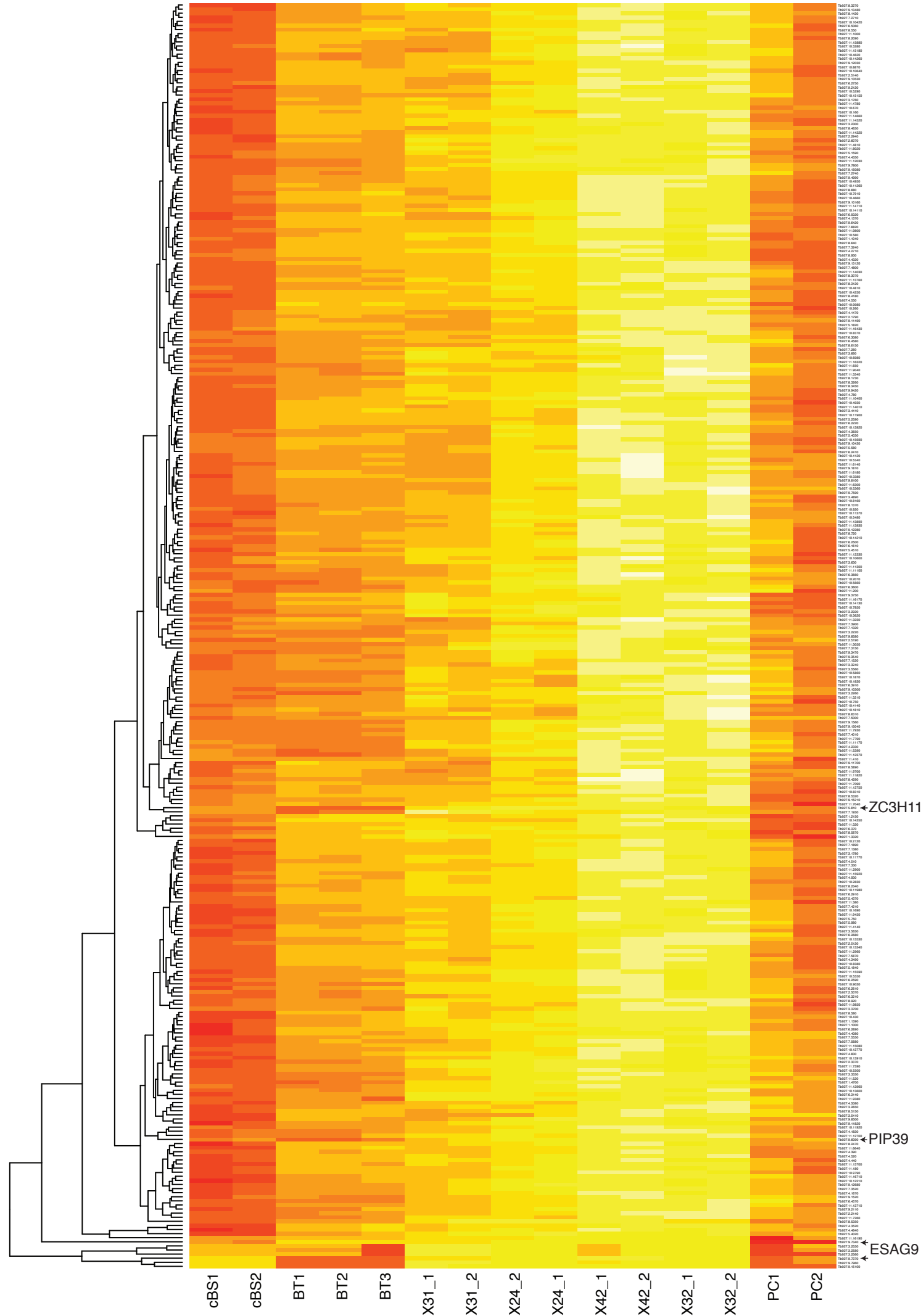

Supplement: Additional file 6: Figure S2. — Heat map of the regulation factors (log2) for mRNAs that are increased in the LW032 and LW042 transcriptomes. Red is least and white is most. (PDF 617 kb) [file 12864_2015_2338_MOESM6_ESM.pdf]

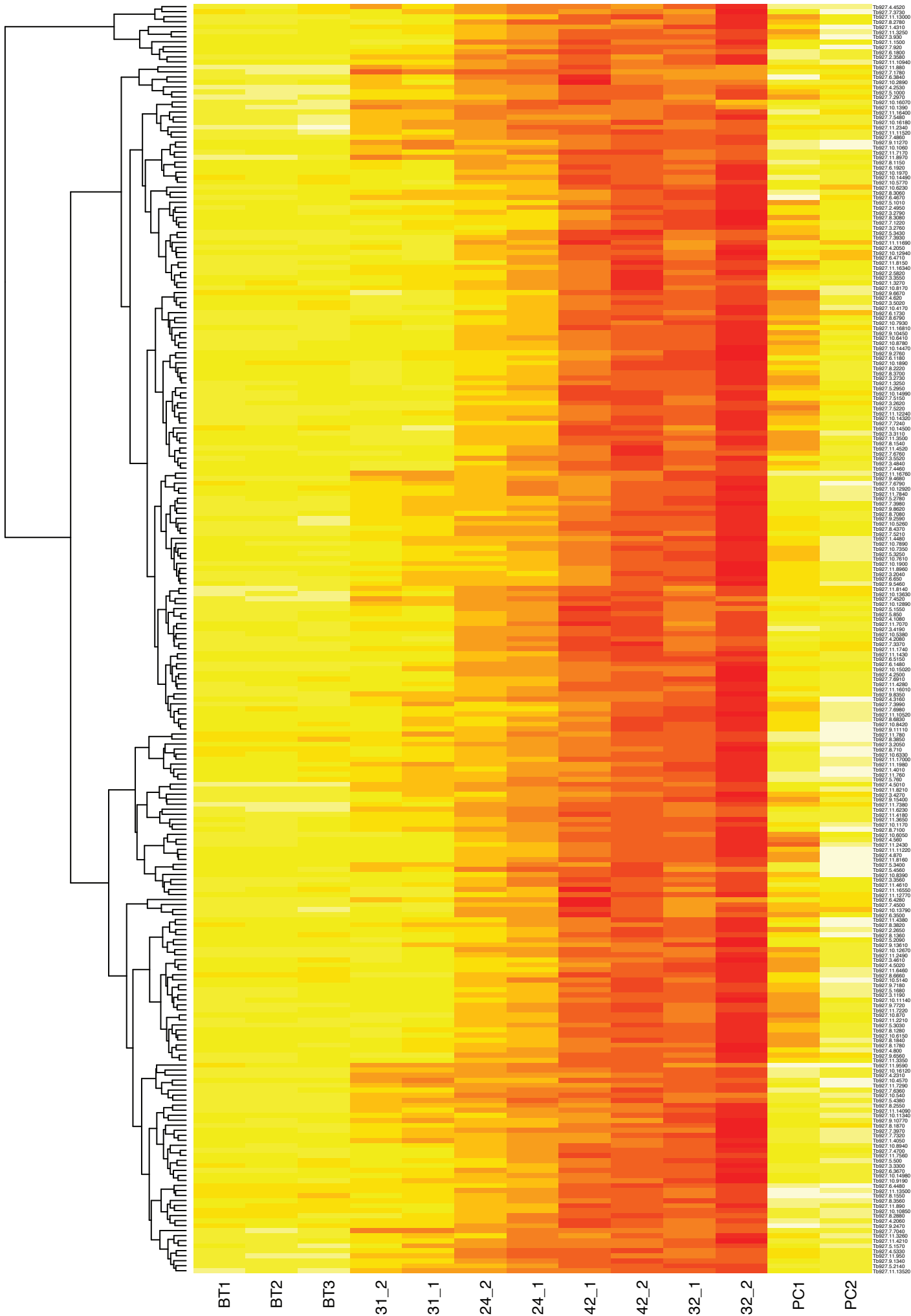

Supplement: Additional file 7: Figure S3. — Heat map of the regulation factors (log2) for mRNAs that are decreased in the LW032 and LW042 transcriptomes. Red is least and white is most. (PDF 570 kb) [file 12864_2015_2338_MOESM7_ESM.pdf]
